# DNA methylation status correlates with adult β-cell regeneration capacity

**DOI:** 10.1038/s41536-021-00119-1

**Published:** 2021-02-12

**Authors:** Ishant Khurana, Keith Al-Hasani, Scott Maxwell, Harikrishnan K.N., Jun Okabe, Mark E. Cooper, Patrick Collombat, Assam El-Osta

**Affiliations:** 1 Department of Diabetes, Central Clinical School, Monash University, Melbourne, VIC Australia; 2 Epigenetics in Human Health and Disease Laboratory, Central Clinical School, Monash University, Melbourne, VIC Australia; 3 Université Côte d’Azur, Inserm, CNRS, iBV, Nice, France; 4 Department of Medicine and Therapeutics, The Chinese University of Hong Kong, Hong Kong, SAR Hong Kong; 5 Hong Kong Institute of Diabetes and Obesity, Prince of Wales Hospital, The Chinese University of Hong Kong, 3/F Lui Che Woo Clinical Sciences Building, 30-32 Ngan Shing Street, Sha Tin, Hong Kong, SAR Hong Kong; 6 Li Ka Shing Institute of Health Sciences, The Chinese University of Hong Kong, Hong Kong, SAR Hong Kong; 7 University College Copenhagen, Faculty of Health, Department of Technology, Biomedical Laboratory Science, Copenhagen, Denmark

**Keywords:** Metabolic disorders, Diabetes

## Abstract

The role of DNA methylation in β-cell neogenesis is poorly understood. We report that during the process of induced cell reprogramming, methylation content of the *Ngn3* and *Sox11* genes are diminished. These findings emphasise DNA methylation is a barrier in β-cell regeneration in adulthood, a well described pathophysiological phenomenon of major significance in explaining β-cell deficiency in diabetes in the adult pancreas.

The development of diabetes involves pathogenetic processes that either destroy the β-cells of the pancreas or result in resistance to insulin action. Type 1 diabetes (T1D) is an autoimmune disease that selectively destroys insulin-producing β-cells in the pancreas. Even though symptoms usually do not appear before 80% of the β-cell mass has been destroyed, absolute destruction of these cells leads to the dependence on exogenous insulin administration for survival. In patients with Type 2 diabetes (T2D), insulin is either produced in insufficient quantities so the response to insulin is weak or it is produced in normal amounts, but the target organs become insulin resistant.

Two solutions aimed at replacing the damaged β-cell mass in diabetic patients exist, such as whole pancreas or islets transplantation. Although efficient, these therapies face the shortage of organ donors together with the associated side-effects of immunosuppressive drugs. Consequently, current research focuses on the replacement of the lost β-cell in diabetic patients using several approaches and cell sources. However, critical to exploiting the potential of these regenerative approaches, is understanding how tissue and cellular processes are controlled during development.

In the pancreas, endocrine cell allocation and maintenance of the different endocrine cell lineages are controlled by transcription factors that precisely regulate glucose homoeostasis. During development, this transcriptional hierarchy itself is in part regulated by epigenetic modifications. The master gene involved in endocrine fate determination is Neurogenin3 (*Ngn3*)^1,2^. *Ngn3* is required for the development of all the endocrine cells (α-, β-, δ-, PP- and ε-cells), that are all associated with the secretion of specific endocrine hormones. Moreover, during pancreas morphogenesis, *Ngn3* induces the delamination of progenitors from the ductal epithelium through an epithelial-to-mesenchymal transition (EMT) process^3^. EMT is a key developmental program by which cells located within an epithelial layer acquire the ability to spread and migrate to a distant site to form new structures mediated by *Sox11*^4^. Ngn3-expressing progenitors, subsequently migrate and emerge from the ductal epithelium and aggregate to eventually form the islets of Langerhans.

Arx and Pax4 are key transcription factors for the specification towards the α-/PP- and β-/δ- cell fates, respectively^5^. Indeed, *Pax4* is critical for β-cell determination and is exclusively expressed in β-cells in the adult pancreas, whereas *Arx* plays a key role in the determination of the α/PP-cell lineage and is restricted to mature glucagon-expressing cells where it is involved in maintaining their identity. In fact, *Arx* and *Pax4* display antagonistic activities with respect to the allocation of the endocrine precursors through an inhibitory cross-regulatory circuit that controls the transcriptional state of these two genes^5^.

A potential source of β-cells was previously demonstrated with the discovery of α-cell plasticity and the ability of α-cell to convert into insulin-producing cells. This is dependent on the ectopic expression of *Pax4* in adult or embryonic α-cells for conversion into β-like cells^6,7^. Conversely, the loss of *Arx* in glucagon-expressing cells triggers their conversion into functional insulin-producing cells^8^. Equally important was the finding that the α- to β-like cell conversion observed in these models induces the re-expression of *Ngn3* in ductal cells and their differentiation into endocrine cells by reawakening EMT.

In this study, we assessed DNA methylation in order to gain a better understanding how this epigenetic mark impacts gene expression during cell reprogramming. We show that in two transgenic mouse models of α-to-β-cell conversion by way of directed transcription factor reprogramming, *Ngn3* and *Sox11* genes undergo dramatic reductions in DNA methylation content which is consistent with re-expression at the mRNA level. Our in vivo studies propose the *Ngn3* and *Sox11* genes are demethylated during adult β-cell regeneration.

The main goal of this study was to determine whether the reactivation potential of *Ngn3* and *Sox11* by way of direct lineage conversion is dependent on DNA methylation. We made use of two transgenic models generated previously by our group^7,8^ in which α-cells are continuously regenerated and converted into functional β-like cells through *Pax4* overexpression (PaxOE) or *Arx* deletion (ArxKO) (Fig. 1a). Both these models conclusively established the ductal and α-cell ontogeny of these transdifferentiated β-cells by direct lineage tracing experiments. We showed that *Ngn3* re-expression is a feature of pancreatic progenitors in the duct, and *Sox11* is a hallmark of EMT (Figs. 1b, c). Indeed, using immunofluorescence, we confirmed the detection of numerous insulin-producing cells co-expressing Ngn3 in islets. *Sox11* was also assessed because DNA demethylation is thought to be an early regulatory event required for epithelial gene reactivation^9^. Both *Ngn3* and *Sox11* were undetected in adult WT mice (data not shown). We isolated DNA and RNA from islets that were purified from mice treated with doxycycline for a period of three months. DNA methylation capture from isolated islets was performed using methylation-capture technique followed by qPCR assessment for the reprogramming (rE) genes *Ngn3* and *Sox11* (Fig. 1d). The UCSC genome browser (mm10) was used to identify CpG rich regions on the promoters of *Ngn3* and *Sox11* (Fig. 1e). *Oct4*, being a stem cell marker, was used as an endogenous control since its expression is not altered during adult lineage reprogramming^7^. The DNA methylation content of the *Ngn3* and *Sox11* promoters were significantly reduced in reprogrammed cells when compared to respective controls (Fig. 1f). We assessed whether the changes we observed for DNA methylation were inversely corelated with *Ngn3* and *Sox11* gene expression in islets derived from the same PaxOE and ArxKO animals. When we compared expression levels from these transgenic lines versus respective control islets, we observed significant increases in *Ngn3* and *Sox11* mRNA levels (Fig. 1g). We also assessed the mechanism by which cell reprogramming reduced DNA methylation influenced the expression of the *Ngn3* and *Sox11* genes. Critical to DNA demethylation, the expression of enzymes of the ten-eleven translocation (TET) family catalyse the stepwise oxidation of 5-methylcytosine in DNA to 5-hydroxymethylcytosine and further oxidation products resulting in the loss of methylation^10^. We therefore assessed the expression of the DNA demethylase family of genes *Tet1*, *Tet2* and *Tet3* and show α- to β-cell trans-differentiation upregulates *Tet* mRNA levels (Fig. 1h). In addition to *Tet2* mRNA upregulation, *Tet1* is also elevated in the ArxKO model while *Tet3* is increased in the Pax4OE. Taken together, these data are consistent with the postulate that the regulatory barrier to transition of pancreatic α-cell to β-cells in an adult regenerative context is dependent on the loss of DNA methylation.

**Fig. 1:**
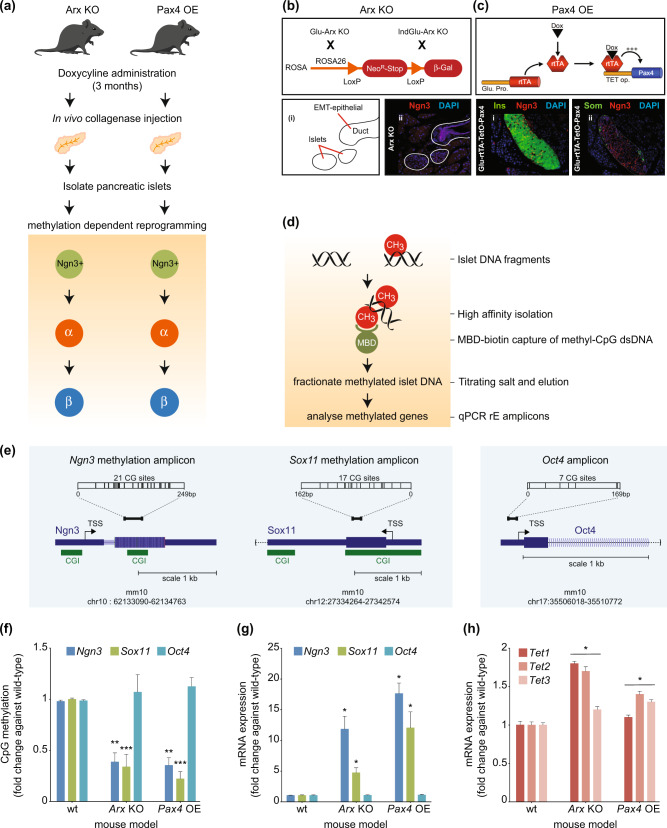
DNA methylation-dependent reprogramming of islet cells derived from Arx knockout and overexpression of Pax4 animal models. **a** Glu-rtTA::TetO-Pax4 animals were generated as previously described [7], treated with Dox at 4 weeks of age for 3 months. The ArxKO mouse line was crossed with Glu-rtTA transgenic line permitting the inducible deletion of *Arx* in adult α-cells [8]. Mice were then treated with Dox for 3 months. Islets from both transgenic lines were then purified to assess for methylation-dependent reprogramming. **b**, **c** Analysis of *Ngn3* re-expression in the mouse pancreata. The expression of *Ngn3* was analysed by immunohistochemistry in WT/Dox- controls and Dox-treated Pax4OE and ArxKO pancreata. *Ngn3* labelling was absent in controls, while strongly re-expressed in induced animals (**b**, **c**). *Ngn3* is re-expressed in the ductal lining and epithelium (**b** *i and ii*) as well as in the islets (**b**, **c**) of transgenic mice while being absent in controls. **d** Workflow of DNA methylation capture and analysis of transgenic mice islets using methyl-domain-binding (MBD) capture and downstream qPCR (MBD-qPCR) were used for the assessment of the reprogramming (rE) amplicons *Ngn3* and *Sox11*. **e** The reprogramming amplicons *Ngn3* and *Sox11* were designed from the mouse genome assembly (mm10) using UCSC browser. CpG Islands (CGI) are shown in green for *Ngn3* and *Sox11*. *Oct4* does not have a CGI and served as a control for DNA methylation. The positions of the transcription start sites (TSS) are also shown relative to 1 kb scale. Chromosome positions are shown *Ngn* (chr10), *Sox11* (chr12) and *Oct4* (chr17). **f** DNA methylation analysis of islet development genes in transgenic mouse models, PaxOE and ArxKO. Data show DNA methylation (fold-change) for *Ngn3* and *Sox11*. A member of the POU transcription factor family, *Oct4*, central to the machinery governing pluripotency served as an endogenous control and remains stable for DNA methylation. Error bars are defined as standard error of the mean (s.e.m) with significance calculated by comparing wild-type (wt) to transgenic mouse models PaxOE and ArxKO, using *t*-test (*P < 0.05, **P < 0.01, ***P < 0.001). **g** mRNA expression of genes associated with islet lineage reprogramming. Data shows gene expression (fold-change) normalised to housekeeping gene (*H3F3A*). SEM error bars with significance calculated by comparing wild-type (wt) to transgenic mouse models PaxOE and ArxKO, (*P < 0.05). **h** mRNA expression of ten eleven translocation (Tet) enzymes in transgenic mice islets. Error bars are defined as standard error of the mean (s.e.m) with significance calculated comparing wild-type (wt) to transgenic mouse models PaxOE and ArxKO, (*P < 0.05).

DNA demethylation is a key condition required to activate developmental genes during islet cell trans-differentiation. Even though DNA methylation is known to correlate with gene expression, it has not previously been shown that this modification is erased from the *Ngn3* and *Sox11* genes for reactivation during adult in vivo cell reprogramming. We have used two independent transgenic models, PaxOE and ArxKO, which mediate conversion of mature α-cells into β-like cells to reveal a regulatory role of DNA methylation during α-cell to β-cell trans-differentiation. Our study connects DNA demethylation, specifically, the expression of the Tet enzymes with the developmental genes *Ngn3* and *Sox11* in the context of islet β-cell regeneration. As previously mentioned, *Ngn3* is a pro-endocrine gene that is only expressed in endocrine progenitors and is thought to control the trans-differentiation process of progenitor cells into endocrine cells. Thus, *Ngn3* appears to be an ideal candidate for strategies that aim to influence DNA demethylation using chemical inhibitors thereby enabling pancreatic β-cell regeneration as a potential path towards improved treatments for T1 and T2 diabetes. A model of DNA demethylation-mediated reprogramming (dmrE) of progenitor cells into β-cells is illustrated and described in Fig. 2. In two distinct transgenic models we observe reduced methylation content of the developmental genes *Ngn3* and *Sox11* are tightly correlated with renewed transcriptional competence. The precise mechanisms of gene regulation during α- to β-cell trans-differentiation is poorly understood. The events associated with *Ngn3* and *Sox11* gene re-expression in ductal precursor cells (Fig. 2a) prior to converting into α-cells and subsequently into β-cells (Fig. 2b) is correlated with reduced DNA methylation (Fig. 2c). The conventional view of silencing is well characterised by reader proteins that recognise and tightly bind genes in a methylation-specific manner^11^. This makes the methylation moiety on genes as effective substrates for reader proteins to assemble onto chromatin and suppress transcription. Secondly, reader proteins also reside in stable complexes that are associated with histone deacetylase activity that function to suppress gene activity on methylated DNA^12^. This would be the most direct mechanism by which methylation interferes with transcription. This mechanism also highlights the complex nature of methylation and its functional importance as a dynamic form of regulation in endocrine cells because transcription is tuned on when the methyl-CG determinant is diminished. The data presented here implicate the loss of methylation during trans-differentiation. We postulate these events are associated with a loss of transcriptional suppressors specific for methylated DNA. In a simple scenario, the capacity to suppress demethylated genes is less effective and replaced with gene-activating regulatory complexes that also influence β-cell capacity. In this scenario, the loss of methylation and gain in *Ngn3* and *Sox11* transcription corresponds with elevated *Tet2* expression during trans-differentiation suggesting the removal of the methylation barrier is important to generate functional newly formed β-cells.

**Fig. 2:**
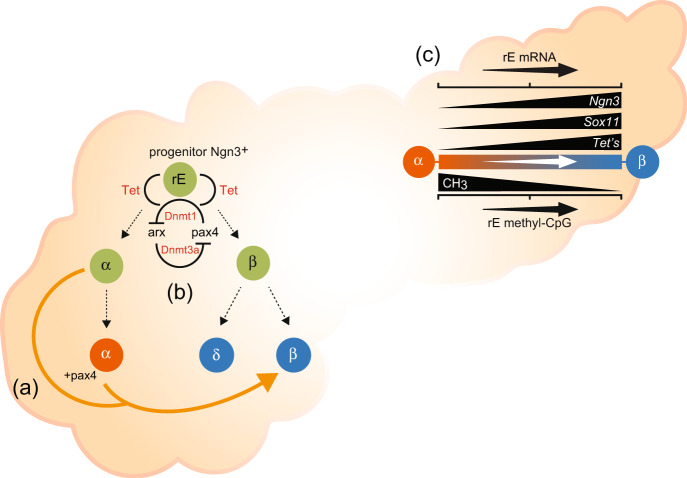
Model of DNA demethylation-mediated reprogramming (dmrE). Proposed schematic model of DNA methylation as an epigenetic barrier for islet transition and reprogramming in the pancreas. **a** *Pax4* overexpression in adult α-cells (orange) induce their trans-differentiation and conversion into β-like cells (blue). This leads to a shortage in glucagon, which is responsible for the mobilisation of ductal precursor cells (green), these re-expressing the pro-endocrine gene *Ngn3* (green), prior to undergoing an EMT and concomitant differentiation into endocrine cells. Such a continuous cycle of conversion/regeneration results in insulin+ cell hyperplasia. **b** Methylation writing (DNMT’s) and erasing (Tet’s) enzymes are implicated in the regulation of *Arx* and *Pax4*, respectively. **c** Re-expression of *Ngn3* is inversely associated with loss of DNA methylation in the ArxKO knockout and *Pax4* misexpression animal models. This model of DNA demethylation-mediated reprogramming or dmrE closely corresponds with *Tet* expression and the loss of gene methylation content and enhanced activity of the mesenchymal marker *Sox11*.

Furthermore, 5-aza-cytidine a pharmacological inhibitor of DNA methylation was previously used in the conversion of adult human skin fibroblasts into insulin-secreting cells indicating this epigenetic mark represents a barrier to reprogramming^13^. Taken together, we found that overexpression of *Pax4* in α-cells or conversely the inducible deletion of *Arx*, influences DNA methylation content in *Ngn3* and *Sox11* expressing cells by elevating *Tet2* expression. Further studies are warranted to address the specific epigenetic mechanisms regulating *Ngn3* expression in a regenerative context. By defining these pathways this knowledge could greatly assist in resolving a major obstacle in regenerating β-cells in adulthood thereby restoring the β-cell mass in pathophysiological conditions such as T1 and T2D.

## Methods

### Mice and animal procedures

Animal care and experimental procedures were conducted according to the French ethical regulations. Animal protocols were reviewed and approved by an institutional ethics committee (Ciepal-Azur) at the University of Nice, and all colonies were maintained following European animal research guidelines. This project received approval from ethics committee (NCE/2011-22, University of Nice). Wild-type (WT) 129/sv mice were obtained from Charles River Laboratories and from Taconic. The bitransgenic Glu-rtTA::TetO-Pax4 mouse line was generated previously by the crossing of two single transgenic lines, Glu-rtTA and TetO-Pax4 that were generated by classical pronuclear injection^7^. The ArxKO transgenic line used to invalidate the *Arx* gene in pancreatic glucagon-producing cells was previously described^7,8^. Doxycycline (Dox; Sigma) was administered in the drinking water at a concentration of 2 g/L and treatment commenced at 4 weeks of age for a duration of 3 months to specifically drive *Pax4* expression and *Arx* inactivation.

### Islet isolation and DNA methylation analysis

Methyl-CpG-binding domain capture was used to investigate DNA methylation in wild-type, ArxKO and PAX4OE mouse models. Genomic DNA (gDNA) was extracted from mouse islets (4 animals-pooled per group from 3 groups, 12 mice). Pancreases were manually disrupted and injected with collagenase (1 mg/ml) directly into the main pancreatic duct quickly after animal death to digest the pancreatic tissue followed by a protein gradient to separate the islets from the rest of the tissue.

Briefly, purified gDNA was fragmented by sonication using the Q800R sonicator (Qsonica); fragmentation was confirmed by capillary electrophoresis on the MultiNA (Shimadzu). 500 ng of fragmented gDNA was used for methyl-CpG enrichment using MethylMiner (Life Technologies) as previously described^14^. Eluted DNA was assessed by quantitative PCR to calculate the percentage or fold change of methylation for each sample by comparing amplification (Ct values) for the target genes using unbound (unmethylated) and bound (methylated) fractions. Primer sequences for mouse mm10 build; NGN3_DNAm_Forward CACTCTCATACCTAGGGACTGCT and NGN3_DNAm_Reverse ATCTTTGTAAGTTTGGCGTCATC (amplicon size 249 bp and contains 21 CpG sites); SOX11_DNAm_Forward AATTCAAGCTCAGGTCGAACAT and SOX11_DNAm_Reverse ACTACAGCTTCAAGAACATCACCA (amplicon size 162 bp and 17 CpG sites); OCT4_DNAm_Forward CGAGCAACTGGTTTGTGAGG and OCT4_DNAm_Reverse GAAACTGAGGCGAGCGCTAT (amplicon size 167 and 7 CpG sites). To assess changes in content, a methylation ratio of genetically modified mice was compared with wild type. Data are shown as mean ± standard error of the mean (SEM). Statistical significance and *P*-values were calculated by 2-tailed, paired Student’s *t*-tests (Graphpad Prism 8).

### Gene expression analysis

Total RNA from mouse pancreatic islets was isolated using TRIzol (Invitrogen) and RNeasy Kit (QIAGEN) including a DNase treatment. The quantification of mRNA levels was performed as previously described^7^. Briefly, quantitative RT-PCR analyses were undertaken using the QuantiTect SYBR Green RT- PCR kit (Roche) and Qiagen primers using a LightCycler 480 instrument (Roche Life Science). Expression levels of specific genes were tested and normalised to housekeeping gene (H3F3A). Each qPCR reaction contained: 5 μl 2x supermix, 0.5 μl PrimerAssay, 3 μl H2O and 1.5 μl of previously synthesised cDNA, diluted 1/20.

### Immunohistochemistry

Immunohistochemistry of Ngn3, insulin and somatostatin was performed as previously described^7^. Briefly, tissues were fixed for 30 min in 4% paraformaldehyde at 4 °C and embedded in paraffin and 8 mm sections applied to slides. Paraffin sections were deparaffinized three times for 3 min in xylene, rehydrated in decreasing ethanol dilutions (5 min in 2 × 95%; 5 min in 80%; 5 min in 60%; 5 min in 30%), and finally rinsed twice for 5 min in ddH2O. The sections were then washed three times for 5 min in PBS and incubated in 10% FCS in PBS for 1 h at RT. The sections were then incubated with the primary antibody appropriately diluted in 10% FCS in PBS overnight at 4 °C in a humid chamber. The primary antibodies used were the following: mouse monoclonal anti-insulin (1/500; Sigma; catalogue #I2018, mouse anti-Ngn3 (1/10,000; Millipore; catalogue #AB5684), rat monoclonal anti-somatostatin (1/250; Sigma; catalogue #MAB354). Pictures were processed using ZEISS Axioimager Z1.

### Reporting summary

Further information on research design is available in the Nature Research Reporting Summary linked to this article.

## Supplementary information

Reporting Summary Checklist

## Data Availability

The data that support the findings of this study are available from the corresponding author upon reasonable request.
